# Concordance between transbronchial lung cryobiopsy and surgical lung biopsy for interstitial lung disease in the same patients

**DOI:** 10.1186/s12890-023-02571-9

**Published:** 2023-07-29

**Authors:** Tomohisa Baba, Tamiko Takemura, Koji Okudela, Akira Hebisawa, Shoichiro Matsushita, Tae Iwasawa, Hideaki Yamakawa, Hiroaki Nakagawa, Takashi Ogura

**Affiliations:** 1 Department of Respiratory Medicine, Kanagawa Cardiovascular and Respiratory Center, Tomioka-Higashi 6-16-1, Kanazawa-Ku, Yokohama, Japan; 2 Department of Pathology, Kanagawa Cardiovascular and Respiratory Center, Yokohama, Japan; 3 Department of Pathology, Graduate School of Medicine, Yokohama City University, Yokohama, Japan; 4 Department of Clinical Research, National Hospital Organization Tokyo National Hospital, Tokyo, Japan; 5 Department of Radiology, Graduate School of Medicine, Yokohama City University, Yokohama, Japan; 6 Department of Radiology, Kanagawa Cardiovascular and Respiratory Center, Yokohama, Japan; 7 Department of Respiratory Medicine, Saitama Red Cross Hospital, Saitama, Japan; 8 Division of Respiratory Medicine, Department of Internal Medicine, Shiga University of Medical Science, Otsu, Japan

**Keywords:** Multidisciplinary discussion, Confidence level, Idiopathic pulmonary fibrosis, Interstitial pneumonia, Cryobiopsy

## Abstract

**Background:**

The diagnostic accuracy and safety of transbronchial lung cryobiopsy (TBLC) via a flexible bronchoscope under sedation compared with that of surgical lung biopsy (SLB) in the same patients is unknown.

**Methods:**

Retrospectively the data of fifty-two patients with interstitial lung diseases (median age: 63.5 years; 21 auto-antibody positive) who underwent TBLC followed by SLB (median time from TBLC to SLB: 57 days) was collected. The samples from TBLC and SLB were randomly labelled to mask the relationship between the two samples. Diagnosis was made independently by pathologists, radiologists, and pulmonary physicians in a stepwise manner, and a final diagnosis was made at multidisciplinary discussion (MDD). In each diagnostic step the specific diagnosis, the diagnostic confidence level, idiopathic pulmonary fibrosis (IPF) diagnostic guideline criteria, and treatment strategy were recorded.

**Results:**

Without clinical and radiological information, the agreement between the histological diagnoses by TBLC and SLB was 42.3% (kappa [κ] = 0.23, 95% confidence interval [CI]: 0.08–0.39). However, the agreement between the TBLC-MDD and SLB-MDD diagnoses and IPF/non-IPF diagnosis using the two biopsy methods was 65.4% (κ = 0.57, 95% CI: 0.42–0.73) and 90.4% (47/52), respectively. Out of 38 (73.1%) cases diagnosed with high or definite confidence at TBLC-MDD, 29 had concordant SLB-MDD diagnoses (agreement: 76.3%, κ = 0.71, 95% CI: 0.55–0.87), and the agreement for IPF/non-IPF diagnoses was 97.4% (37/38). By adding the pathological diagnosis, the inter-observer agreement of clinical diagnosis improved from κ = 0.22 to κ = 0.42 for TBLC and from κ = 0.27 to κ = 0.38 for SLB, and the prevalence of high or definite diagnostic confidence improved from 23.0% to 73.0% and from 17.3% to 73.0%, respectively. Of all 383 TBLC performed during the same period, pneumothorax occurred in 5.0% of cases, and no severe bleeding, acute exacerbation of interstitial lung disease, or fatal event was observed.

**Conclusions:**

TBLC via a flexible bronchoscope under deep sedation is safely performed, and the TBLC-MDD diagnosis with a high or definite confidence level is concordant with the SLB-MDD diagnosis in the same patients.

**Supplementary Information:**

The online version contains supplementary material available at 10.1186/s12890-023-02571-9.

## Summary at a glance

Transbronchial lung cryobiopsy via a flexible bronchoscope under deep sedation is safely performed, and the multidisciplinary discussion diagnosis of transbronchial lung cryobiopsy with a high or definite confidence level is concordant with that of surgical lung biopsy in the same patients.

## Background

Surgical lung biopsy (SLB) is considered the gold standard for obtaining pathological specimens of diffuse lung disease [1, 2]. However, the risk of mortality after SLB was reported to be 1.7% [3], and even in specialised interstitial lung disease centres, missing histological assessment accounted for approximately half of the patients categorised as unclassifiable interstitial lung disease due to comorbidities, respiratory function impairment, and unwillingness to undergo surgery [4].

Transbronchial lung cryobiopsy (TBLC), which is a relatively new technique to obtain larger and better-preserved specimens, has a higher diagnostic yield than forceps biopsy [5]. TBLC was reported to be slightly inferior to SLB in terms of histological diagnostic yield (82.8% vs. 98.7%) but superior in terms of safety (mortality rate: 0.3% vs. 2.7%) [6]. Similar to SLB, TBLC increases diagnostic confidence in the multidisciplinary diagnosis of idiopathic pulmonary fibrosis (IPF) [7]. Recent IPF clinical practice guideline has conditionally recommended TBLC as an acceptable alternative to SLB in centres with appropriate expertise[8]. However, there have been a few studies directly comparing TBLC and SLB within the same population. Two prospective studies demonstrated the concordance between the TBLC diagnosis and SLB diagnosis in the same patients with interstitial lung disease [9, 10], but the results of these two studies were conflicting. Moreover, the experimental procedures in one operation under general anaesthesia using a rigid bronchoscope, was different from the practical diagnostic procedures performed under sedation using a flexible bronchoscope and conventional endotracheal tube which maintains the convenience, safety, and comfort of TBLC [11]. Another prospective study using flexible bronchoscope through the endobronchial tube demonstrated good concordance between the two biopsy approaches, but the procedure was performed under general anesthesia and the sample was small [12]. As these studies were designed such that TBLC was followed by SLB in one operation, the safety analysis of the TBLC was not possible and TBLC might be done without fear of complications, resulting in high diagnostic yield of the TBLC.

The purpose of this retrospective study was to clarify the concordance between the diagnosis using TBLC performed under sedation with a flexible bronchoscope and that using SLB in patients with interstitial lung disease and the safety of TBLC.

## Methods

### Patients

**Fig. 1:**
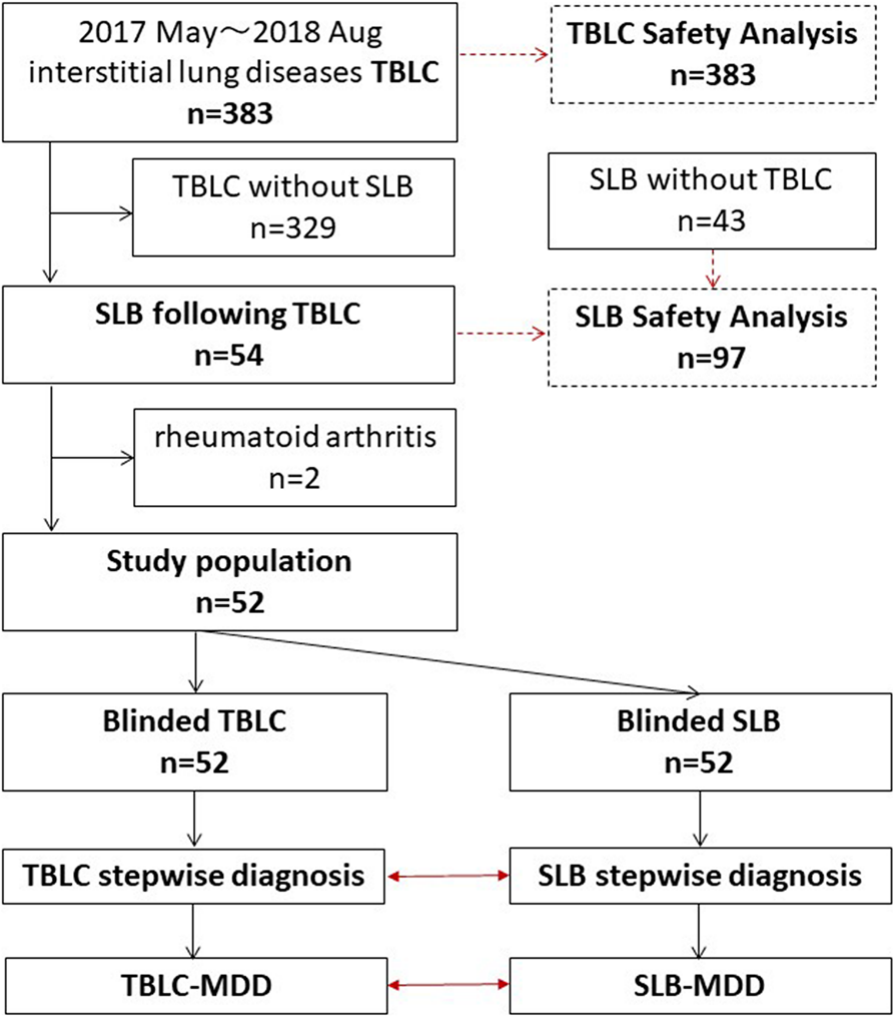
The study flowchart. Among 383 patients who underwent TBLC, 54 underwent sequential SLB. Two patients were diagnosed with rheumatoid arthritis before TBLC. A total of 52 patients were included in this study. The samples from TBLC and SLB were randomly labelled to mask the relationship between the two samples. The diagnosis was made in a stepwise manner and the concordance between the two biopsy approaches was analyzed

This retrospective study included 52 patients with interstitial lung diseases, who underwent TBLC followed by SLB at the Kanagawa Cardiovascular & Respiratory Centre between May 2017 and August 2018. In the clinical diagnostic course, the decision to proceed with SLB following TBLC depended on the physicians’ assessment or the local multidisciplinary discussion (MDD) according to the diagnostic guidelines. In this study patients diagnosed with interstitial lung disease with specific known causes, such as connective tissue diseases, hypersensitivity pneumonitis, and occupational lung diseases before TBLC were excluded (Fig. 1). No pharmacological treatment was performed between the two biopsies. Clinical information, including age at biopsy, sex, smoking history, pulmonary or extrapulmonary signs and symptoms, laboratory data including results of auto-antibody tests, and adverse events of each biopsy, was obtained from the patients’ medical records. For safety analysis, adverse events in all 383 and 97 patients who underwent TBLC and SLB, respectively, during the study period were collected.

### Procedures

For TBLC, patients were intubated using a flexible endotracheal tube under deep sedation with midazolam and fentanyl, and spontaneous breathing was maintained. A 1.9 mm or 2.4 mm cryoprobe (Erbe Elektromedizin, Tübingen, Germany) was inserted through the working channel of a flexible bronchoscope BF-1TQ290 (Olympus Corporation, Tokyo, Japan) and placed into a subpleural location 1 cm from the pleura under fluoroscopic guidance. The 1.9 mm and 2.4 mm probes were activated for 6–7 s and 4–5 s, respectively. The frozen lung parenchyma, cryoprobe, and flexible bronchoscope were removed en bloc, and the samples were placed in formalin. A prophylactic balloon catheter was placed in the targeted airways and inflated after each procedure until haemostasis was achieved [13]. Depending on oxygenation, sedation, and bleeding conditions, up to four specimens were obtained from different segments. Bronchoalveolar lavage was performed during the procedure. The patient was discharged the next day, after confirming the absence of pneumothorax on a chest x-ray.

SLB was performed by thoracic surgeons using video-assisted thoracoscopic surgery under general anaesthesia. In most cases, two samples were obtained from different lobes which were not exactly the same lobes as those sampled by TBLC. After SLB, a drain tube was placed in all patients, they were monitored overnight in the intensive care unit, and discharged from the hospital within several days.

### Diagnosis

The samples from TBLC and SLB were randomly labelled to mask the relationship between the two samples, and the diagnosis was made in a stepwise manner (Supplementary Fig. 1) [14]. In step 1P, three expert pathologists (TT, KO, and HA) independently examined the TBLC and SLB samples without clinical and radiological information. A consensus was reached regarding 1) specific pathological diagnosis, including usual interstitial pneumonia-IPF, non-specific interstitial pneumonia, organising pneumonia, diffuse alveolar damage, desquamative interstitial pneumonia, respiratory bronchiolitis, pleuroparenchymal fibroelastosis, lymphocytic interstitial pneumonia, hypersensitivity pneumonitis (HP), connective tissue disease, fibrosing organising pneumonia, smoking related pneumonia, other specific disease, unclassifiable (e.g. combination) and “not diagnostic”; 2) diagnostic confidence level (definite, high, low, not diagnostic); and 3) IPF diagnostic guideline criteria (definite usual interstitial pneumonia, probable usual interstitial pneumonia, indeterminate for usual interstitial pneumonia, and alternative diagnosis) [15].

In step 1R, high-resolution computed tomography (HRCT) scans at the time of each biopsy were independently reviewed by two experienced radiologists (T.I. and S.M.) without knowledge of the clinical and pathological information and relationship between the HRCT scans. The radiologists made the radiological diagnosis with a diagnostic confidence level and classified the HRCT patterns according to the IPF diagnostic guideline criteria [15]. Disagreements between the two radiologists after the first assessment were resolved by discussion.

Two experienced pulmonary physicians (H.Y. and H.N.) independently made the clinical diagnosis and recorded the diagnostic confidence level and treatment strategy in each diagnostic step without knowing the relations between the TBLC and SLB samples. Even if the clinical diagnosis was “unclassifiable” according to the classification of the idiopathic interstitial pneumonias[2], the diagnostic confidence level could be labelled definite, high, or low when a specific clinical diagnosis was made as fibrosing organising pneumonia, smoking-related pneumonia, or a combination. The diagnosis was made using clinical data and HRCT images in step 1C; the radiological diagnosis, results of the bronchoalveolar lavage analysis when available, and pathological diagnosis were added as single disciplinary diagnoses in steps 2, 3, and 4, respectively. Finally, the multi-disciplinary discussion (MDD) was held with seven experts and MDD diagnosis was reached. Masked paired cases were not linked in each diagnostic step until the statistical analysis was finalized.

### Statistical analysis

Kappa concordance coefficients and percentage agreement (both with their 95% confidence intervals [CIs]) were analysed for individual or consensus TBLC versus SLB diagnosis in each diagnostic step, and for inter-observer TBLC or SLB diagnosis. A κ value ≤ 0.20 indicated poor agreement, 0.21–0.40 indicated fair agreement, 0.41–0.60 indicated moderate agreement, 0.61–0.80 indicated good agreement, and 0.81–1.00 indicated excellent agreement. Basic data are expressed as numbers and medians with interquartile ranges. Statistical analyses were performed using JMP (version 12.2.0 2015; SAS Institute Inc., Cary, NC, USA) and R (version 3.22.3517.0; The R Foundation for Statistical Computing, Vienna, Austria).

## Results

Among 383 patients who underwent TBLC between May 2017 and August 2018 in our respiratory centre, 54 underwent sequential SLB in the same period. Two patients were diagnosed with rheumatoid arthritis before TBLC; therefore, a total of 52 patients were included in this study. Clinical characteristics of the 52 patients and the number of biopsies are summarised in Table 1. The median patient age was 63.5 years (interquartile range; 55.0–67.3), with 22 (42.3%) women and 33 (63.5%) ever smokers. In the serological analysis, 20 (38.5%) were positive for autoantibodies, defined as interstitial pneumonia with autoimmune features serologic domain by Fischer et al. [16] and one patient was positive for myeloperoxidase-anti-neutrophil cytoplasmic antibody. The median time from TBLC to SLB was 57 days. The number of TBLC samples was one in 6 patients, two in 33 patients, and three in 13 patients. In most patients, two samples were obtained through SLB. Only 17% of TBLC samples were obtained from different lobes; in contrast, 94% of SLB samples were obtained from different lobes.

**Table 1:** Clinical characteristics

Gender ( Female / Male)		22 / 30
Age at TBLC (yr)		63.5 (55.0–67.3)
Smoking history (never/ex-smoker/ current)		19 / 30 / 3
Serologic domain of IPAF (yes/no)		20 / 32
%FVC		84.8% (76.6–94.6)
%DLco		69.3% (60.4–80.7)
HRCT IPF diagnostic criteria (UIP/Probable/Indeterminate/Alternative)	TBLC	2/6/21/23
	SLB	3/5/21/23
Time from cryobiopsy to SLB (days)		57 (43.5–81.8)
Numbers of specimens (1/2/3)	TBLC	6 / 33 / 13
	SLB	3 / 47/ 2
Biopsied from multiple lobes (yes/no)	TBLC	9/43
	SLB	49/3

Data are presented as numbers and median (interquartile range)

*IPAF* Interstitial Pneumonia with Autoimmune Features

**Fig. 2:**
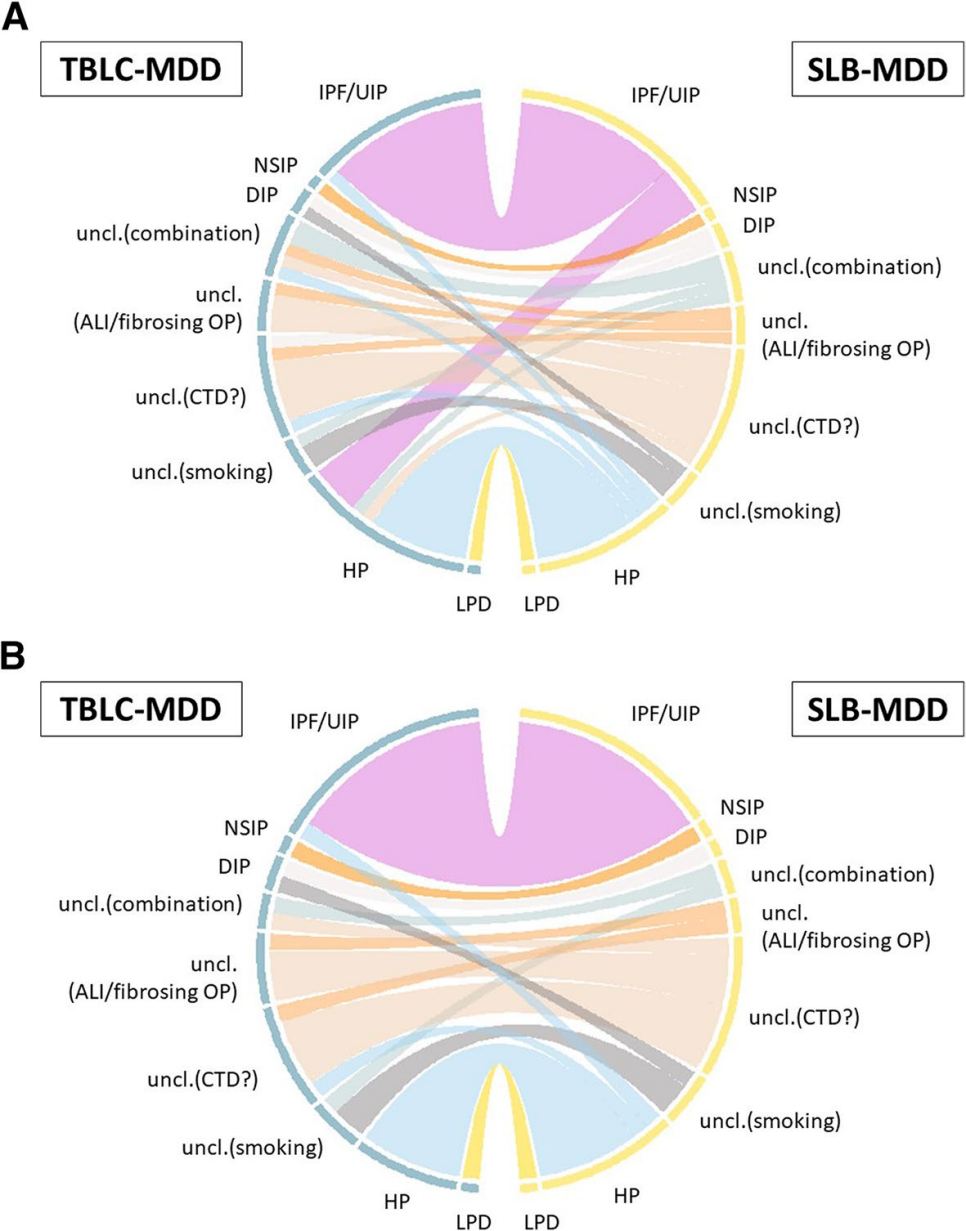
Concordance between TBLC and SLB in the MDD diagnosis. **A**: Concordance between TBLC-MDD diagnosis and SLB-MDD diagnosis in all 52 patients. The concordance between the TBLC-MDD and SLB-MDD diagnoses at step 4 was 65.4% in agreement and 0.57 in kappa (95% CI 0.42–0.73). **B**: 38 TBLC-MDD cases with high or definite confidence. The diagnoses of 29 were concordant with those at SLB-MDD (agreement: 76.3%, κ = 0.71, 95% CI: 0.55–0.87). Abbreviations; ALI/FOP: acute lung injury/fibrosing organising pneumonia, CTD: connective tissue disease, HP: hypersensitivity pneumonitis, LPD: lymphoproliferative disorder, MDD: multidisciplinary discussion, SLB: surgical lung biopsy, TBLC: transbronchial lung cryobiopsy, uncl.: unclassifiable interstitial lung disease, uncl.(smoking): unclassifiable interstitial lung disease(smoking related pneumonia)

The concordance between the diagnosis using TBLC and that using SLB in each diagnostic step is shown in Table 2. At consensus without clinical and radiological information, the concordance between the pathological diagnosis using TBLC and that using SLB was fair (step 1P, agreement: 42.3%, κ = 0.23, 95% CI: 0.08–0.39). For IPF diagnostic guideline-defined histopathological pattern, 4, 15, 11, and 19 samples were classified as definite usual interstitial pneumonia, probable usual interstitial pneumonia, indeterminate for usual interstitial pneumonia, alternative diagnosis with TBLC, 5, 6, 29, and 12 samples were classified as definite, probable, indeterminate, and alternative with SLB, respectively. In three cases, TBLC samples were not sufficient for diagnosis. The agreement for IPF diagnostic guideline-defined histopathological pattern between TBLC and SLB was 38.5% with a κ = 0.19 (95% CI: 0.04–0.33). Finally, the concordance between the TBLC-MDD and SLB-MDD diagnoses at step 4 was 65.4% in agreement and 0.57 in kappa (95% CI 0.42–0.73). Of the 52 TBLC cases, 38 (73.1%) were diagnosed with high or definite confidence at MDD. Of these 38 TBLC-MDD cases, the diagnoses of 29 were concordant with those at SLB-MDD (agreement: 76.3%, κ = 0.71, 95% CI: 0.55–0.87). The relationship between TBLC-MDD diagnoses and SLB-MDD diagnoses for each patient is shown in Fig. 2 (Supplement Table 1). The two most common TBLC-MDD and SLB-MDD diagnoses were IPF and HP. A total of 92.9% (13/14) of patients diagnosed with IPF at TBLC-MDD were diagnosed with IPF at SLB-MDD, and one was diagnosed with HP. On the other hand, 76.5% (13/17) of patients diagnosed with IPF at SLB-MDD were diagnosed with IPF at TBLC-MDD, and four were diagnosed with HP with low confidence. The agreement of IPF/non-IPF diagnoses between the two biopsy methods was 90.4% (47/52) in all patients and 97.4% (37/38) in 38 cases with high or definite confidence TBLC-MDD diagnoses. The concordance of the consensus treatment strategy between TBLC and SLB at step 4 was 82.7% in agreement and 0.74 in kappa (95% CI: 0.59–0.89) (Supplementary Table 2). In addition, the treatment strategies of 18 out of 20 patients diagnosed with unclassifiable interstitial pneumonia at TBLC-MDD matched with those at SLB-MDD.

**Table 2:** Concordance between the consensus diagnosis with TBLC and the diagnosis with SLB

Diagnostic Step	step1P (*n* = 52)	step1R (*n* = 52)	step1C (*n* = 52)	step2 (*n* = 52)	step3 (*n* = 52)	step4 (*n* = 52)	step4(H/D) (*n* = 38)
Agreement	42.3%	53.8%	67.3%	65.3%	55.8%	65.4%	76.3%
kappa	0.23	0.45	0.61	0.59	0.57	0.57	0.71
95%CI	0.08–0.39	0.29–0.61	0.45–0.76	0.44–0.74	0.43–0.72	0.42–0.73	0.55–0.87

Step4 (H/D): 38 cases were diagnosed with high or definite confidence in TBLC-MDD.step1P: consensus pathological diagnosis without clinical and radiological information. step1R: consensus radiological diagnosis without clinical and pathological information. Step1C: Consensus clinical diagnosis without radiological diagnosis and pathological information Step2: Consensus clinical diagnosis with radiological diagnosis and without pathological information Step3: Consensus clinical diagnosis with radiological diagnosis and bronchoalveolar lavage analysis without pathological information Step4: MDD diagnosis with full information, including pathological diagnosis

**Fig. 3:**
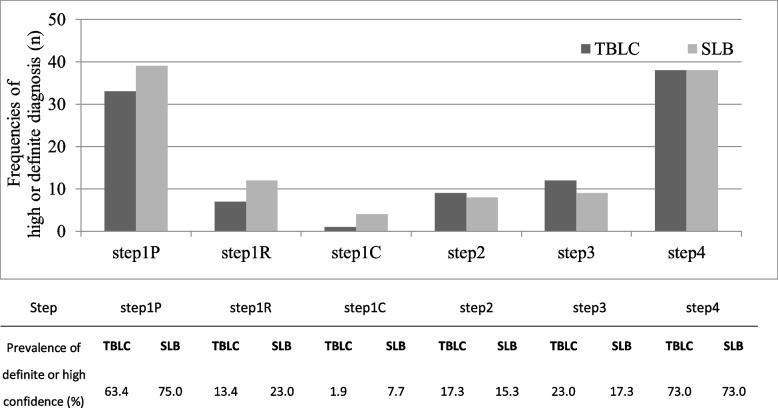
Prevalence of high or definite confidence level of diagnosis at each diagnostic step

When comparing steps 3 and 4, by adding the pathological diagnosis, the inter-observer agreement of clinical diagnosis improved from κ = 0.22 to κ = 0.42 in TBLC, and from κ = 0.27 to κ = 0.38 in SLB (Table 3). Similarly, the prevalence of high or definite diagnostic confidence level increased from 23.0% to 73.0% in TBLC and from 17.3% to 73.0% in SLB, which shows that the pathological diagnosis using TBLC had the same impact on the diagnostic confidence in MDD of interstitial lung disease as that using SLB (Fig. 3). In addition, the inter-observer agreement for treatment strategy rose from κ = 0.36 to κ = 0.59 in TBLC, and from κ = 0.38 to κ = 0.53 in SLB (Supplementary Table 3).

**Table 3:** Inter-observer agreement of the diagnosis in TBLC and SLB in each diagnostic step

Diagnostic Step	step1P (A vs B)		step1P (C vs A)		step1P (B vs C)		step1R (S vs T)		step1C (X vs Y)		step2 (X vs Y)		step3 (X vs Y)		step4 (X vs Y)	
	TBLC	SLB	TBLC	SLB	TBLC	SLB	TBLC	SLB	TBLC	SLB	TBLC	SLB	TBLC	SLB	TBLC	SLB
Agreement	63.4	42.3	55.8	57.7	63.5	50.0	44.2	32.7	32.7	36.5	32.7	34.6	32.7	36.5	51.9	48.1
kappa	0.50	0.24	0.42	0.45	0.51	0.31	0.30	0.22	0.21	0.25	0.23	0.25	0.22	0.27	0.42	0.38
95%CI	0.34–0.65	0.10–0.39	0.26–0.57	0.28–0.62	0.35–0.66	0.15–0.47	0.15–0.46	0.08–0.35	0.07–0.35	0.12–0.39	0.10–0.36	0.13–0.37	0.08–0.36	0.14–0.39	0.28–0.56	0.24–0.52

A, B, and C refer to each pathologist, S and T radiologist, and X, Y pulmonary physician

The adverse events in all 383 TBLC and 97 SLB cases during the study period are shown in Supplementary Table 4. Pneumothorax occurred in 5.0% (19/383) of TBLC; in 6 cases (1.6%), single aspiration or drainage was performed, and no case required surgery. In contrast, 3.1% (3/97) of patients who underwent SLB experienced prolonged air leak after surgery, and two (2.0%) required revision surgery. Although moderate airway bleeding managed with local haemostatic agents occurred in 16.4% (63/383) of patients who underwent TBLC, no severe bleeding was experienced due to the prophylactic use of the balloon catheter. Neither TBLC nor SLB caused acute exacerbation of interstitial lung disease or other fatal events during this period.

## Discussion

Our study demonstrates good concordance between the TBLC-MDD and SLB-MDD diagnoses in the same patient, especially in cases with high or definite diagnostic confidence in TBLC-MDD diagnoses. To our knowledge, our study is the largest study to compare blinded specimens obtained through TBLC using a flexible bronchoscope under deep sedation and SLB in the same patient in the course of clinical diagnosis.

Without clinical and radiological information, the pathological diagnosis using TBLC and SLB was poorly concordant (κ = 0.23), similar to that in Romagnoli’s study (21 patients, κ = 0.22) [9]. One possible reason for this is the difference in biopsy sites between TBLC and SLB. The target region of TBLC is proximal lung tissue approximately 1 cm from the pleura, in contrast to peripheral lung tissue containing pleura obtained with SLB. Another reason is that without clinical and radiological information, the inter-observer concordance of pathological diagnosis is low even with SLB [17, 18] . Therefore, poor concordance between pathological diagnosis using TBLC and SLB is unavoidable. Moreover, the inter-observer agreement was better for TBLC pathological diagnosis than for SLB with high diagnostic confidence. Since TBLC specimens are smaller than SLB specimens, and contain fewer findings, pathologists may be able to diagnose without hesitation. Therefore, multiple TBLC specimens from different lobes are desirable [19, 20] and MDD is mandatory for the diagnosis of diffuse lung disease [21].

In the COLDICE study, agreement for the specific histopathological pattern identified by pathologists for paired TBLC and SLB was 69.2% with κ = 0.47 (95% CI: 0.30–0.64) and diagnostic agreement at MDD was 76.9% (κ = 0.62, 95% CI 0.47–0.78). The possible reasons for the slightly lower agreement in our study are as follows. First, although the median number of biopsies was 5 in the COLDICE study and increased numbers of TBLC samples were reported to predict histopathologic concordance with SLB [22], the median number biopsied with TBLC was 2 in our study. As same as in COLDICE study, higher agreement rates were observed in higher numbers of biopsied samples (The diagnostic concordance between TBLC-MDD and SLB-MDD was 50% for one TBLC sample, 64% for two samples and 75% for three samples, respectively). Low numbers biopsied is a limitation of using a flexible bronchoscope under deep sedation compared with the rigid bronchoscope used under general anaesthesia in the COLDICE study. Besides, in our study the biopsy cites were not exactly same between the two techniques, which may limit the agreement of TBLC-MDD and SLB-MDD[23]. Second, because the design of the COLDICE study included TBLC followed by SLB, TBLC might have been performed without fear of complications from multiple regions near the pleura, resulting in a high diagnostic yield of TBLC. In our study, pneumothorax occurred in 5.0% of cases, which was lower than the pooled analysis rate of 13.4% [15].Thus, the exact region biopsied may have been distant from the pleura, and samples may have contained proximal lung tissue with bronchial walls, resulting in a lower diagnostic yield of TBLC. Because TBLC in this study was performed immediately after approval of TBLC in Japan, improving the procedure and sedation may increase the number of biopsies or biopsy sites (from multiple lobes) and increase the diagnostic confidence level. Third, some TBLC cases which were diagnosed with high confidence in MDD might not undergo SLB in practice. For this selection bias, it may be difficult to diagnose TBLC cases in this study with high confidence. Finally, selectable diagnostic categories were more in this study than in the COLDICE study, and unclassified interstitial pneumonia was subdivided according to specific causes. Therefore, the agreement between TBLC-MDD and SLB-MDD was inevitably reduced.

Although 20 of 52 TBLC cases were diagnosed as unclassified interstitial pneumonia in MDD, the treatment strategy of these 20 cases matched the strategy derived from SLB-MDD. This result demonstrates the usefulness of TBLC-MDD in making treatment decisions, even if a guideline-based specific diagnosis is not reached.

TBLC has been reported to have a meaningful impact on diagnostic confidence in the MDD diagnosis of interstitial lung disease in previous studies [7, 24]. Diagnostic confidence is a subjective standard, but diagnosis using TBLC with MDD is reliable if the diagnostic confidence level is “definite” or “high”. In such cases, the agreement of MDD diagnoses between the biopsy methods was 76.3% (κ = 0.71) and the agreement for IPF/non-IPF diagnoses was 97.4% (37/38). It may be practical approach to perform SLB, re-challenge TBLC or assess clinical behaviour in case of “low diagnostic confidence” or “not diagnostic” in TBLC-MDD[25]. This stepwise diagnostic approach can maintain a reliable diagnostic yield with the convenience and low cost of flexible bronchoscopy and minimise the adverse events of SLB.

In this study, TBLC was performed without severe complications using a flexible bronchoscope under deep sedation in the endoscopy room. Since flexible bronchoscopy is more common than rigid bronchoscopy, TBLC with a flexible bronchoscope has the advantage of decreasing unclassified interstitial pneumonia due to the lack of pathological samples.

There were several limitations to this study that should be considered when interpreting our results. First, the TBLC and SLB samples evaluated in this study were obtained from a single centre. However, it was considered difficult to study across multiple institutes because SLB is rarely performed following TBLC in clinical settings. Second, although clinical and radiological information and biopsied samples were anonymized, and the relation between the two data sets was masked during diagnosis, pulmonologists may have identified the relationship of clinico-radiological information due to their memories. To avoid such concerns, the progression interval between diagnostic steps was sufficiently long to wash out memories. Since SLB-MDD was essential for diagnosis, at step 4, SLB-MDD was performed after completing all steps of TBLC-MDD so that SLB-MDD did not influence TBLC-MDD.

## Conclusion

MDD is mandatory for the diagnosis of interstitial lung disease and TBLC-MDD diagnosis with high or definite confidence is concordant with SLB-MDD diagnosis in the same patients.

## Supplementary Information

**Additional file 1: Supplementary Figure 1. **Flowchart of the study design. **Supplementary Table 1. **Concordance between TBLC-MDDdiagnosis and SLB-MDD diagnosis at step 4. A: Data for all 52 patients, B: Batafor 38 TBLC-MDD cases with high or definite confidence at step 4. **Supplement Table 2.** Concordance of consensustreatment strategies between TBLC-MDD and SLB-MDD. **Supplement Table 2.** Concordance of consensustreatment strategies between TBLC-MDD and SLB-MDD. **Supplement Table 4. **Adverseevents related to the procedure. 

## Data Availability

The datasets generated and/or analyzed during the current study are not publicly available due to study participant privacy/consent agreements but are available from the corresponding author on reasonable request.

## Abbreviations

ALI/FOP: Acute lung injury/fibrosing organising pneumonia
ATS: American Thoracic Society
BALF: Bronchoalveolar lavage fluid
CTD: Connective tissue disease
DIP: Desquamative interstitial pneumonia
%DLco: Percentage of predicted diffusing capacity for carbon monoxide
ERS: European Respiratory Society
%FVC: Percentage of predicted forced vital capacity
HP: Hypersensitivity pneumonitis
HRCT: High-resolution computed tomography
IIP: Idiopathic interstitial pneumonia
ILD: Interstitial lung disease
IP: Interstitial pneumonia
IPAF: Interstitial pneumonia with autoimmune features
IPF: Idiopathic pulmonary fibrosis
KL-6: Krebs von Lungen 6
MDD: Multidisciplinary discussion
LPD: Lymphoproliferative disorder
NSIP: Non-specific interstitial pneumonia
PPFE: Pleuroparenchymal fibroelastosis
SLB: Surgical lung biopsy
SP-D: Surfactant protein D
TBLC: Transbronchial lung cyrobiopsy
UIP: Usual interstitial pneumonia
Uncl.: Unclassifiable interstitial lung disease
