# Supplementary material for: Concordance between transbronchial lung cryobiopsy and surgical lung biopsy for interstitial lung disease in the same patients
Source: BMC Pulm Med. 2023 Jul 29;23:279. doi: 10.1186/s12890-023-02571-9 (PMC10385958; doi:10.1186/s12890-023-02571-9)
Supplement: Supplementary file 1 — Additional file 1: Supplementary Figure 1. Flowchart of the study design. Supplementary Table 1. Concordance between TBLC-MDDdiagnosis and SLB-MDD diagnosis at step 4. A: Data for all 52 patients, B: Batafor 38 TBLC-MDD cases with high or definite confidence at step 4. Supplement Table 2. Concordance of consensustreatment strategies between TBLC-MDD and SLB-MDD. Supplement Table 2. Concordance of consensustreatment strategies between TBLC-MDD and SLB-MDD. Supplement Table 4. Adverseevents related to the procedure. [file 12890_2023_2571_MOESM1_ESM.docx]

**Online Data Supplement**

**Concordance between transbronchial lung cryobiopsy and surgical lung biopsy for interstitial lung disease in the same patients**

Tomohisa Baba, Tamiko Takemura, Koji Okudela, Akira Hebisawa, Shoichiro Matsushita, Tae Iwasawa, Hideaki Yamakawa, Hiroaki Nakagawa and Takashi Ogura

**Supplementary Figure 1**

Flowchart of the study design

Samples were anonymized, so that relations between the two samples were not identified, and were diagnosed in a stepwise manner. A, B, and C represent pathologists, S and T radiologists, and X, Y pulmonary physicians. Black arrows indicate concordance analysis between the diagnoses. Although consensus diagnoses were also made in steps 1, 2, and 3, and concordance between the consensus diagnoses of TBLC and SLB data sets were analysed, these are not shown in this flow chart.

TBLC: transbronchial lung cryobiopsy, SLB: surgical lung biopsy


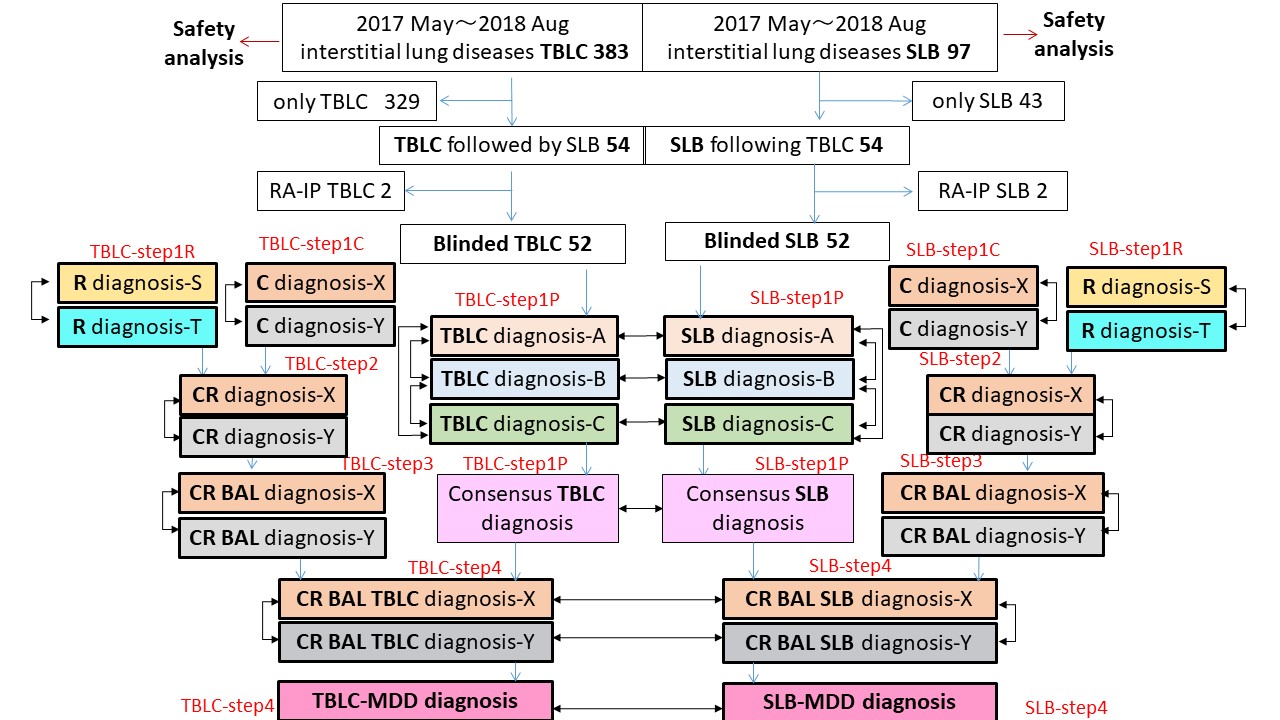


**Supplementary Tables**

**Supplementary Table 1. Concordance between TBLC-MDD diagnosis and SLB-MDD diagnosis at step 4. A: Data for all 52 patients, B: Bata for 38 TBLC-MDD cases with high or definite confidence at step 4**

**A) All 52 patients**

|  |  | **SLB-MDD diagnosis** | | | | | | | | | |  |
| --- | --- | --- | --- | --- | --- | --- | --- | --- | --- | --- | --- | --- |
|  |  | IPF/UIP | NSIP | DIP | uncl.  (combination) | uncl.  (ALI/FOP) | uncl.  (CTD?) | uncl.  (smoking) | HP | LPD | Total | |
| **TBLC-MDD diagnosis** | IPF/UIP | 13 | 0 | 0 | 0 | 0 | 0 | 0 | 1 | 0 | 14 | |
|  | NSIP | 0 | 1 | 0 | 0 | 0 | 0 | 0 | 0 | 0 | 1 | |
|  | DIP | 0 | 0 | 1 | 0 | 0 | 0 | 1 | 0 | 0 | 2 | |
|  | uncl.  (combination) | 0 | 0 | 0 | 2 | 1 | 1 | 0 | 1 | 0 | 5 | |
|  | uncl.  (ALI/FOP) | 0 | 0 | 0 | 0 | 1 | 3 | 0 | 0 | 0 | 4 | |
|  | uncl.  (CTD?) | 0 | 0 | 1 | 0 | 1 | 5 | 0 | 1 | 0 | 8 | |
|  | uncl.  (smoking) | 0 | 0 | 0 | 1 | 0 | 0 | 2 | 0 | 0 | 3 | |
|  | HP | 4 | 0 | 0 | 1 | 0 | 1 | 0 | 8 | 0 | 14 | |
|  | LPD | 0 | 0 | 0 | 0 | 0 | 0 | 0 | 0 | 1 | 1 | |
|  | Total | 17 | 1 | 2 | 4 | 3 | 10 | 3 | 11 | 1 | 52 | |

Agreement: 65.4%, kappa: 0.57 (95% CI: 0.42-0.73). uncl.: unclassifiable interstitial lung disease, TBLC: transbronchial lung cryobiopsy, SLB: surgical lung biopsy, MDD: multidisciplinary discussion, ALI/FOP: acute lung injury/fibrosing organising pneumonia, CTD?: suspected connective tissue disease, HP: hypersensitivity pneumonitis, LPD: lymphoproliferative disorder

**B)** **TBLC-MDD cases with high or definite confidence in at step 4 (n=38)**

|  |  | **SLB-MDD diagnosis** | | | | | | | | | |  |
| --- | --- | --- | --- | --- | --- | --- | --- | --- | --- | --- | --- | --- |
|  |  | IPF/UIP | NSIP | DIP | uncl.  (combination) | uncl.  (ALI/FOP) | uncl.  (CTD?) | uncl.  (smoking) | HP | LPD | Total | |
| **TBLC-MDD diagnosis** | IPF/UIP | 12 | 0 | 0 | 0 | 0 | 0 | 0 | 1 | 0 | 13 | |
|  | NSIP | 0 | 1 | 0 | 0 | 0 | 0 | 0 | 0 | 0 | 1 | |
|  | DIP | 0 | 0 | 1 | 0 | 0 | 0 | 1 | 0 | 0 | 2 | |
|  | uncl.  (combination) | 0 | 0 | 0 | 1 | 0 | 1 | 0 | 0 | 0 | 2 | |
|  | uncl.  (ALI/FOP) | 0 | 0 | 0 | 0 | 1 | 3 | 0 | 0 | 0 | 4 | |
|  | uncl.  (CTD?) | 0 | 0 | 0 | 0 | 1 | 4 | 0 | 1 | 0 | 6 | |
|  | uncl.  (smoking) | 0 | 0 | 0 | 1 | 0 | 0 | 2 | 0 | 0 | 3 | |
|  | HP | 0 | 0 | 0 | 0 | 0 | 0 | 0 | 6 | 0 | 6 | |
|  | LPD | 0 | 0 | 0 | 0 | 0 | 0 | 0 | 0 | 1 | 1 | |
|  | Total | 12 | 1 | 1 | 2 | 2 | 8 | 3 | 8 | 1 | 38 | |

Agreement: 76.3%, kappa: 0.71 (95% CI: 0.55-0.87).

TBLC: transbronchial lung cryobiopsy, SLB: surgical lung biopsy, MDD: multidisciplinary discussion, ALI/FOP: acute lung injury/fibrosing organising pneumonia, CTD?: suspected connective tissue disease, HP: hypersensitivity pneumonitis, LPD: lymphoproliferative disorder

**Supplement Table 2.** **Concordance of consensus treatment strategies between TBLC-MDD and SLB-MDD**

|  |  | SLB-MDD | | | |  |
| --- | --- | --- | --- | --- | --- | --- |
|  |  | Anti-fibrotics | Antigen avoidance | Corticosteroids +/- immunosuppressants | Observation | Total |
| TBLC-MDD | Anti-fibrotics | 17 | 1 | 0 | 0 | 18 |
|  | Antigen avoidance | 5 | 8 | 1 | 0 | 14 |
|  | Corticosteroids +/- immunosuppressants | 1 | 0 | 18 | 1 | 20 |
|  | Observation | 0 | 0 | 0 | 0 | 0 |
|  | Total | 23 | 9 | 19 | 1 | 52 |

Agreement: 82.7%, kappa: 0.74 (95% CI: 0.59-0.89).

**Supplement Table 3. Inter-observer agreement of treatment strategies after TBLC and SLB**

|  | stpe1C | | stpe2 | | stpe3 | | stpe4 | |
| --- | --- | --- | --- | --- | --- | --- | --- | --- |
|  | TBLC | SLB | TBLC | SLB | TBLC | SLB | TBLC | SLB |
| Agreement | 51.9 | 57.7 | 55.8 | 61.5 | 57.7 | 57.7 | 73.1 | 69.2 |
| kappa | 0.27 | 0.37 | 0.33 | 0.44 | 0.36 | 0.38 | 0.59 | 0.53 |
| 95%CI | 0.09-0.45 | 0.18-0.56 | 0.14-0.51 | 0.25-0.62 | 0.18-0.54 | 0.20-0.57 | 0.41-0.77 | 0.34-0.72 |

**Supplement Table 4. Adverse events related to the procedure**

| 2017May-2018Aug | TBLC  (n=383) | SLB*  (n=97) |
| --- | --- | --- |
| Pneumothorax after TBLC  or prolonged air leak after SLB | 19(5.0%) | 3(3.1%) |
| Single puncture or drainage | 6(1.6%) | 0(0.0%) |
| Operation | 0 | 2(2.0%) |
| Hemorrhage |  |  |
| Local hemostatic agent | 63(16.4%) | 0 |
| Acute exacerbation | 0 | 0 |
| Admission to the ICU | 0 | 97(100%) |
| Mortality due to adverse event | 0 | 0 |
| Non diagnostic pattern(pathology) | 21(5.5%) | 0(0.0%) |

*All patients were managed in an intensive care unit after SLB for careful monitoring with a drainage tube.
